# Ligature of the External Iliac Artery and Vein—Rapid Recovery

**Published:** 1858-01

**Authors:** 


*Ligature of the External Iliac Artery and Vein—Rapid Recovery.*—Dr. E. S. Cooper, of San Francisco, relates (Trans. State Med. Soc. Cal.) a case of successful ligature of both the external iliac artery and vein. The disease was a traumatic femoral aneurism. The operation for tying the external iliac was performed in the usual way; but on attempting to separate the peritoneum, which was very much thickened and adherent to adjacent parts, a gush of venous blood occurred from the iliac vein. The only remedy was the ligature, and this was accordingly applied above and below the wound; the artery was then tied. The patient made a good recovery. One fact worthy of note is, that the temperature of the limb did not fall materially. Dr. Cooper adds, that he has ligated the external iliac artery in six dogs, one of which died; in five he tied both iliac artery and vein; all recovered. In the first class, the limb became cold in every instance, in spite of stimulating applications; the sensibility of the limb was also greatly impaired. In the latter class, the heat and sensibility of the limb remained nearly natural from the first.—*Ib.*

